# Microbial Succession of Anaerobic Chitin Degradation in Freshwater Sediments

**DOI:** 10.1128/AEM.00963-19

**Published:** 2019-08-29

**Authors:** Susanne Wörner, Michael Pester

**Affiliations:** a Department of Biology, University of Konstanz, Constance, Germany; b Leibniz Institute DSMZ—German Collection of Microorganisms and Cell Cultures, Brunswick, Germany; c Institute of Microbiology, Technical University of Braunschweig, Brunswick, Germany; University of California, Davis

**Keywords:** *N*-acetylglucosamine, ammonia release, anaerobic degradation, biopolymer, carbon cycle, chitin, lake sediment, next-generation amplicon sequencing, polysaccharides, turnover rate

## Abstract

Chitin is massively produced by freshwater plankton species as a structural element of their exoskeleton or cell wall. At the same time, chitin does not accumulate in the predominantly anoxic sediments, underlining its importance as carbon and nitrogen sources for sedimentary microorganisms. We studied chitin degradation in littoral sediment of Lake Constance, Central Europe’s third largest lake. Turnover of the chitin analog methyl-umbelliferyl-*N*,*N*-diacetylchitobioside (MUF-DC) was highest in the upper oxic sediment layer, with 5.4 nmol MUF-DC h^−1^ (g sediment [dry weight])^−1^. In the underlying anoxic sediment layers, chitin hydrolysis decreased with depth from 1.1 to 0.08 nmol MUF-DC h^−1^ (g sediment [dry weight])^−1^. Bacteria involved in chitin degradation were identified by 16S rRNA (gene) amplicon sequencing of anoxic microcosms incubated in the presence of chitin compared to microcosms amended either with *N*-acetylglucosamine as the monomer of chitin or no substrate. Chitin degradation was driven by a succession of bacteria responding specifically to chitin only. The early phase (0 to 9 days) was dominated by *Chitinivibrio* spp. (*Fibrobacteres*). The intermediate phase (9 to 21 days) was characterized by a higher diversity of chitin responders, including, besides *Chitinivibrio* spp., also members of the phyla *Bacteroidetes*, *Proteobacteria*, *Spirochaetes*, and *Chloroflexi*. In the late phase (21 to 43 days), the *Chitinivibrio* populations broke down with a parallel strong increase of *Ruminiclostridium* spp. (formerly *Clostridium* cluster III, *Firmicutes*), which became the dominating chitin responders. Our study provides quantitative insights into anaerobic chitin degradation in lake sediments and linked this to a model of microbial succession associated with this activity.

**IMPORTANCE** Chitin is the most abundant biopolymer in aquatic environments, with a direct impact on the carbon and nitrogen cycles. Despite its massive production as a structural element of crustaceans, insects, or algae, it does not accumulate in sediments. Little is known about its turnover in predominantly anoxic freshwater sediments and the responsible microorganisms. We proved that chitin is readily degraded under anoxic conditions and linked this to a succession of the members of the responsible microbial community over a 43-day period. While *Fibrobacteres* and *Firmicutes* members were driving the early and late phases of chitin degradation, respectively, a more diverse community was involved in chitin degradation in the intermediate phase. Entirely different microorganisms responded toward the chitin monomer *N*-acetylglucosamine, which underscores that soluble monomers are poor and misleading substrates to study polymer-utilizing microorganisms. Our study provides quantitative insights into the microbial ecology driving anaerobic chitin degradation in freshwater sediments.

## Abstract (precis)

Chitin is the most abundant biopolymer in aquatic environments, with a direct impact on the carbon and nitrogen cycles. Despite its massive production as a structural element of crustaceans, insects, or algae, it does not accumulate in sediments. Little is known about its turnover in predominantly anoxic freshwater sediments and the responsible microorganisms. We proved that chitin is readily degraded under anoxic conditions and linked this to a succession of the members of the responsible microbial community over a 43-day period. While *Fibrobacteres* and *Firmicutes* members were driving the early and late phases of chitin degradation, respectively, a more diverse community was involved in chitin degradation in the intermediate phase. Entirely different microorganisms responded toward the chitin monomer *N*-acetylglucosamine, which underscores that soluble monomers are poor and misleading substrates to study polymer-utilizing microorganisms. Our study provides quantitative insights into the microbial ecology driving anaerobic chitin degradation in freshwater sediments.

## INTRODUCTION

In freshwater environments, chitin is an important and abundant biopolymer with an estimated annual production of 28 × 10^6^ tons (1, 2). Chitin serves as a structural element of the exoskeleton of arthropods (e.g., crustaceans within the zooplankton and aquatic insects), the cell wall of fungi, and certain algae and protozoa (1). Its polymeric structure consists of linked amino-sugar subunits with the disaccharide *N*,*N*-diacetylchitobiose (GlcNAc)_2_ as the structural subunit and β-1,4-*N*-acetyl-d-glucosamine (GlcNAc) as the monomeric compound. Despite its high annual production, chitin does not accumulate in nature (3). This efficient turnover (50% to 75%) is also observed in freshwater sediments (3) and identifies chitin as an important carbon and nitrogen source for microorganisms in these habitats (1, 2).

Cultured microorganisms that hydrolyze chitin and use it as a growth substrate under oxic conditions are found within the *Betaproteobacteria*, *Gammaproteobacteria*, Deltaproteobacteria, *Firmicutes*, and *Actinobacteria*, whereas most anaerobic chitinolytic microorganisms are found within the *Clostridia* and *Fibrobacteres* (4–8). However, chitinase genes have also been found to be carried by members of the *Acidobacteria*, *Bacteroidetes*, *Cyanobacteria*, *Spirochaetes*, and *Chloroflexi* (9, 10). Chitin depolymerization occurs first by cleavage of the polymer into water-soluble oligomers, followed by splitting of these oligomers into dimers and finally of the dimers into monomers. This process involves endo- and exochitinases. Endochitinases randomly hydrolyze chitin, releasing a mixture of oligomers of different lengths for further depolymerization. Exochitinases remove mono- or disaccharides from the nonreducing ends of chitin oligomers or chitin. In addition, β-*N*-acetylhexosaminidases (chitobiosidase) hydrolyze the dimer *N*,*N*-diacetylchitobiose (1, 9, 11).

Microbial polymer degradation is best understood for cellulose, but the high structural similarity of chitin and cellulose likely results in similar steps of the chitinolytic and cellulolytic pathways. Under oxic conditions, bacteria secrete a mixture of soluble hydrolyzing enzymes that act synergistically on the polymer (12). Under anoxic conditions, microorganisms (mainly *Clostridia*) were shown to be attached to the polymeric fiber and produce large multienzyme complexes that are cell associated outside the cell (13). These complexes are well studied in cellulolytic microorganisms, but investigations of such enzymatic systems are still missing in chitinolytic microorganisms (14, 15). The cell-bound enzyme complexes prevent cross-feeding of breakdown products and enable at the same time a much higher turnover rate than a combination of different secreted enzymes. Most of these hydrolytic microorganisms transport oligomers into the cell (16), which are than cleaved to monomeric structures and metabolized. This cleavage is often mediated by phosphorylases rather than by hydrolytic cleavage, conserving the energy in the linkage (17–20). Another mechanism of anaerobic polymer turnover was observed in the phylum *Fibrobacteres*, with all cultured members being specialized for anaerobic polymer degradation (21–23). These bacteria also attach to the polymeric substance and even lose their ability to hydrolyze the polymer if the association is disturbed. *Fibrobacteres* members of the genera *Chitinivibrio* and *Chitinispirillum* are specialists of chitin degradation, with *Chitinivibrio* spp. even being unable to use polymeric chains shorter than (GlcNAc)_6_. All members transport polymer chains of different lengths through the outer membrane into the periplasmic space, where these oligomers are cleaved and subsequently metabolized in the cell (8, 24).

Environmental studies on chitin degradation focused mainly on soils and peatlands under oxic conditions. Here, members of the *Betaproteobacteria*, *Gammaproteobacteria*, *Acidobacteria*, and *Bacteroidetes* were shown to be involved in chitin degradation (25–27). Chitin degradation under anoxic conditions is less well studied. In anoxic slurries of agricultural soil, which were investigated using *chiA* (encoding chitinase A) as a functional marker gene, a large diversity of *chiA* with unknown affiliation was detected but also *chiA* related to *Betaproteobacteria* and *Gammaproteobacteria* (28). In contrast, a study focusing on anoxic wetland soil also identified members of the *Firmicutes*, *Acidobacteria*, *Verrucomicrobia*, *Alphaproteobacteria*, *Betaproteobacteria*, *Gammaproteobacteria*, and *Deltaproteobacteria* that respond specifically to chitin amendment (29). In this study, we investigated the microbial community responsible for the turnover of chitin and its monomer GlcNAc under anoxic conditions in littoral sediment of oligotrophic Lake Constance. We combined activity assays of chitin turnover with bacterial 16S rRNA gene and 16S rRNA cDNA amplicon sequencing and showed that anaerobic chitin degradation occurs readily and is mediated by a succession of different microbial taxa.

## RESULTS

### Chitin is readily hydrolyzed in oxic and anoxic sediment layers.

**FIG 1:**
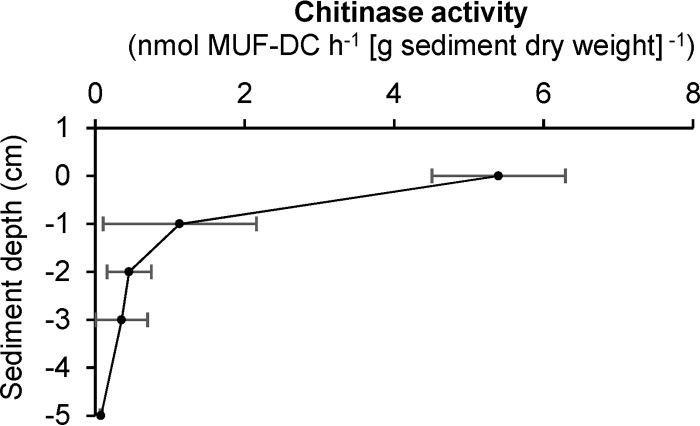
Depth profile of chitinase activities in littoral sediment of Lake Constance. Chitin hydrolysis rates were determined by measuring the turnover of the chitin analog methyl-umbelliferyl-*N*,*N*-diacetylchitobioside (MUF-DC). Mean and one standard deviation (*n* = 3) are shown for each sediment depth.

Chitinase activity can be measured in both oxic and anoxic sediment layers by following the turnover of the chitin analog methyl-umbelliferyl-*N*,*N*-diacetylchitobioside (MUF-DC). Highest chitinase activities were measured in the upper oxic sediment layer with 5.4 nmol MUF-DC h^−1^ (g sediment [dry weight])^−1^ (Fig. 1). In anoxic sediment layers, chitin hydrolysis rates showed a decreasing depth profile. Highest activity was measured 1 cm below the surface with an activity of 1.1 nmol MUF-DC h^−1^ (g sediment [dry weight])^−1^ and a decreasing hydrolysis rate down to 5 cm below the surface with a chitinase activity of 0.08 nmol MUF-DC h^−1^ (g sediment [dry weight])^−1^. A one-factorial analysis of variance (ANOVA) revealed significant differences within the overall depth profiles of chitinase activity (F_4,10_ = 36.04, *P* < 0.001). *Post hoc* two-sample *t* tests identified that the observed differences in chitinase activity between the oxic and all anoxic layers were significant (Benjamini and Hochberg false-discovery rate [FDR]-adjusted *P* < 0.05), while the observed decrease within the anoxic sediment layers was not significant (Benjamini and Hochberg FDR-adjusted *P* > 0.05).

**FIG 2:**
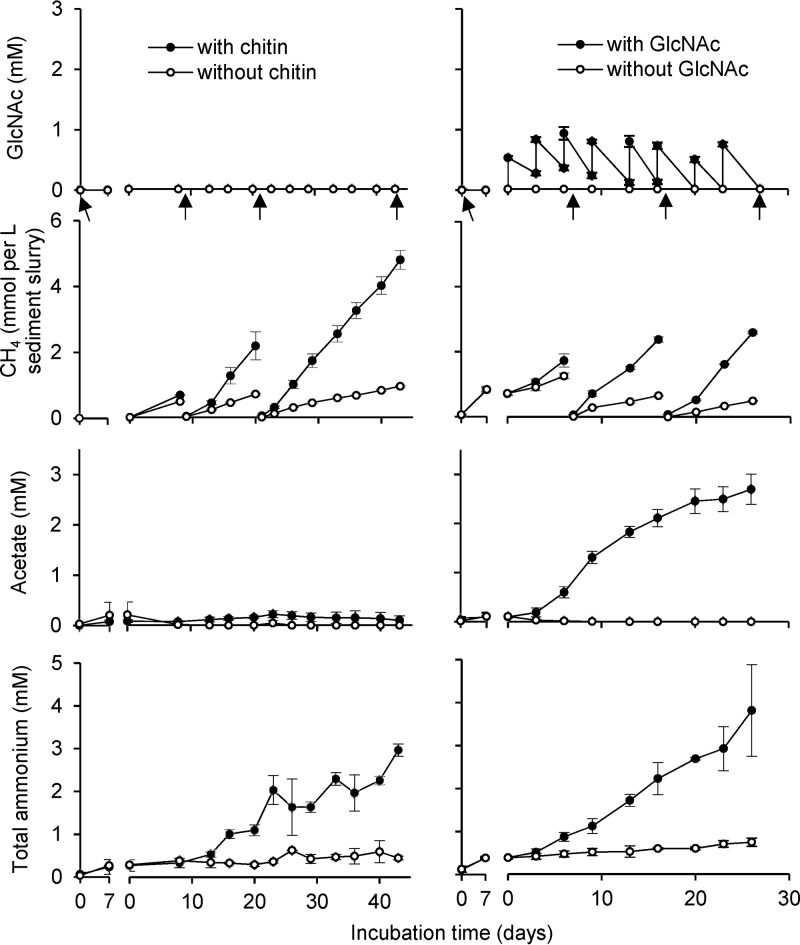
Time course of substrate amendment and product formation in chitin- or GlcNAc-amended microcosms and their respective controls. The gap after the first 7 days represents the shift from preincubation to the single chitin or first GlcNAc amendment. Arrows indicate days of sediment sampling. Mean and one standard deviation (*n* = 3) are shown. Some error bars are smaller than the symbol size.

In further experiments, we concentrated on chitin degradation under anoxic conditions. Anoxic sediment microcosms were used to investigate the degradation of chitin and its monomer GlcNAc. Ammonium, measured as an indicator for amino sugar degradation, accumulated to 2.8 mM in the chitin-amended treatments, which was significantly higher than in the respective control, where only 0.4 mM was measured (*P* < 0.01). The difference in accumulated ammonium indicated a total chitin turnover of 2.4 mM (142 μmol) within the 43 days of incubation (Fig. 2, Table 1). This translated into a turnover rate of 4.4 nmol chitin h^−1^ (g sediment [dry weight])^−1^. Accumulation of GlcNAc due to chitin hydrolysis was not detected in chitin-amended microcosms. Among the tested substances butyrate, propionate, lactate, acetate, and formate, chitin turnover resulted only in a small accumulation of acetate, with a concentration of 97 ± 87 μM at the end of the incubation, which, however, was not significantly different from the control (*P* > 0.1). CH_4_ accumulated in all microcosms, which was 3.6-fold higher in the chitin-amended microcosms than in the respective control without external substrate (*P* < 0.01), (Fig. 2). An electron balance of the estimated chitin hydrolysis (32 e^−^ per GlcNAc-equivalent) revealed a recovery of 60% in CH_4_ (8 e^−^ per molecule) and 1% in acetate (8 e^−^ per molecule) (Table 1).

**TABLE 1:** Overview of the sum of supplemented substrates and recovered products at the end of the four different incubation setups

Substrate or product	Amt recovered (μmol) after incubating:^*a*^			
	With chitin	Without chitin	With GlcNAc	Without GlcNAc
Total amended amino sugars	678		283 ± 11	
Acetate	6 ± 5	ND^*b*^	148 ± 16	ND
Propionate	ND	ND	6 ± 1	ND
Hydrogen	ND	ND	ND	ND
Methane	459 ± 42	128 ± 4	464 ± 22	166 ± 8
Total ammonium	167 ± 8	25 ± 2	229 ± 64	45 ± 6

a For each setup, the averages and standard deviations from three replicates are shown.

b ND, not detected.

In GlcNAc-amended microcosms, periodically supplied GlcNAc was readily consumed without apparent delay within 3 to 4 days. This translated to an average GlcNAc degradation rate of 14.0 nmol GlcNAc h^−1^ (g sediment [dry weight])^−1^. Degradation resulted in an accumulation of acetate to 2.7 ± 0.3 mM toward the end of the incubation, which was significantly different from the respective controls without external substrate, in which no acetate accumulated toward the end of the incubation time (*P* < 0.01). Traces of propionate with an end concentration of 100 ± 26 μM were measured in the GlcNAc-amended microcosms as well. CH_4_ was again a major degradation product, which showed a 4.3-fold higher accumulation in GlcNAc-amended microcosms than in the respective controls without external substrate (*P* < 0.01) (Fig. 2). Although ammonium accumulated steadily as well, only 65% was recovered, as would be expected from the total GlcNAc turnover (Table 1). Nevertheless, the observed accumulation toward the end of the incubation was significantly higher than the respective control without external substrate (*P* < 0.05). An electron balance of the degraded GlcNAc (32 e^−^ per molecule) revealed a recovery of 26% in CH_4_ (8 e^−^), 14% in acetate (8 e^−^ per molecule), and 1% in propionate (14 e^−^ per molecule) (Table 1).

### Chitin amendment altered a minor portion of bacterial beta diversity.

At the onset of the microcosm experiment, the sediment harbored 14,262 ± 496 (mean ± standard deviation) bacterial species-level operational taxonomic units (OTUs) when rarefied to an even sequencing depth of 68,826 reads per replicate (see Fig. S2 in the supplemental material) and 1.2 ± 0.4 × 10^8^ 16S rRNA gene copies (g sediment [dry weight])^−1^ (see Fig. S3). Both parameters stayed rather stable throughout the incubation period. At the end of the individual incubation lines, measured alpha diversity was still at 12,797 ± 754 OTUs (Fig. S2). Also, total 16S rRNA gene copy numbers remained highly stable within 17 and 21 days for GlcNAc- and chitin-amended treatments, respectively. At the end of the incubation time, GlcNAc-amended treatments revealed a slight increase (9.5 × 10^8^ ± 2.8 × 10^8^) and chitin-amended treatments a slight decrease (6.2 × 10^7^ ± 0.3 × 10^7^) in 16S rRNA gene copy numbers (Fig. S3). Good’s coverage revealed that on average, 91% of the bacterial alpha diversity was covered with the following phyla each representing more than 1% relative abundance of the detected 16S rRNA genes: *Proteobacteria* (classes *Alphaproteobacteria*, *Betaproteobacteria*, *Gammaproteobacteria*, and *Deltaproteobacteria*), *Bacteroidetes*, *Chloroflexi*, *Actinobacteria*, *Acidobacteria*, *Verrucomicrobia*, *Planctomycetes*, *Aminicenantes*, *Cyanobacteria/Chloroplast*, *Ignavibacteriae*, and unclassified bacteria. In the GlcNAc incubation line, *Firmicutes* and *Parcubacteria* were, in addition, part of the highly abundant initial microbial community (see Table S1).

**FIG 3:**
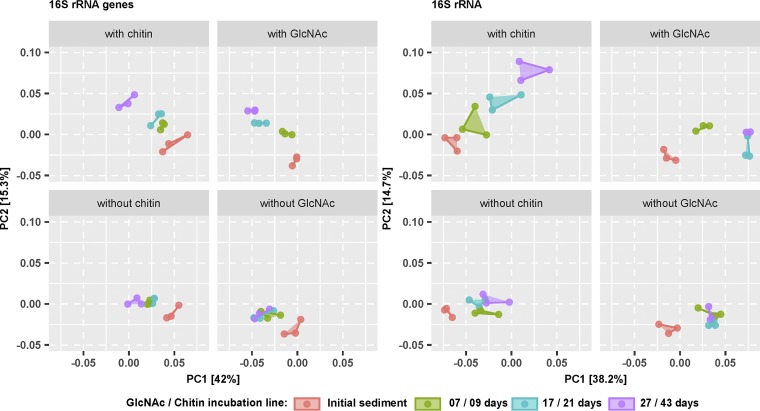
Time-resolved beta diversity of bacterial communities in the various microcosm setups according to a principal coordinate analysis (PCoA) based on the weighted UniFrac metric. Segregation of the bacterial community over time is shown for the 16S rRNA gene and 16S rRNA amplicon survey separately. Connected points of the same color represent biological replicates (*n* = 3).

Temporal changes in beta diversity were analyzed using the weighted UniFrac metric (30) and visualized by a principal-coordinate analysis (PCoA). Starting communities were highly similar within the individual incubation lines, both at the 16S rRNA and 16S rRNA gene levels. However, between the chitin and GlcNAc incubation lines, a slightly different bacterial community was evident, which likely mirrored the different starting sediments (Fig. 3). In all treatments, a slight segregation occurred after the onset of the experiment. Thereafter, the bacterial communities in the chitin and GlcNAc controls (no substrate addition) showed no further separation of the bacterial community over time, both on the 16S rRNA and 16S rRNA gene levels. On the contrary, substrate-amended microcosms showed a continuous separation between all sampling days from the initial community at both the 16S rRNA gene and 16S rRNA levels. Differences between the chitin- and GlcNAc-amended microcosms were more pronounced at the 16S rRNA than at the 16S rRNA gene level (Fig. 3). The observed shifts in community composition were significant (*P* < 0.05) in both substrate-amended incubation lines as revealed by a permutational analysis of variance (PERMANOVA). In chitin-amended microcosms, 28% (31%) of the variation was explained by incubation time and 12% (10%) by chitin addition at the 16S rRNA (gene) level. Similarly, in GlcNAc-amended microcosms, 26% (28%) was explained by time and 19% (16%) by GlcNAc addition at the 16S rRNA (gene) level.

### Chitin degradation triggers a succession of different responders.

**FIG 4:**
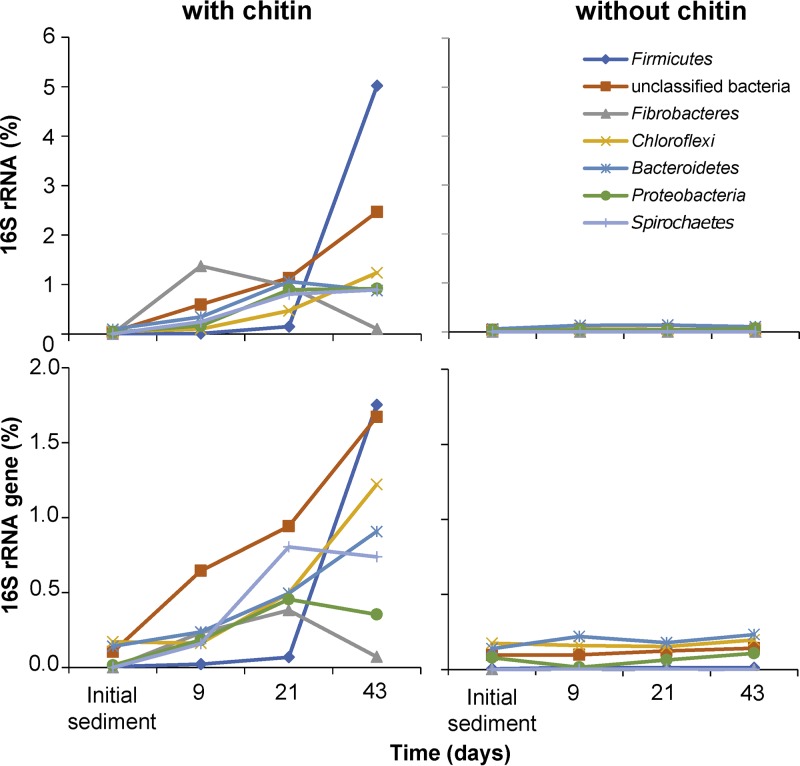
Temporal changes of OTUs (summarized by phylum) at the 16S rRNA level that significantly responded (FDR-corrected *P* < 0.05) to chitin over time compared to the no-substrate control. Only phyla that corresponded in sum of their responding OTUs to ≥0.9% of bacterial 16S rRNA copies are shown for clarity. For details, please refer to Table S2 in the supplemental material.

OTUs responsible for chitin-specific differences in beta diversity were identified by significant changes at the 16S rRNA or 16S rRNA gene level in response to chitin treatment over time. Of the total bacterial community, 203 OTUs representing 13 phyla and unclassified bacteria showed a significant response toward chitin amendment within the 43 days of incubation (see Table S2). In sum, responding OTUs steadily increased from 0.5% to 12.6% at the 16S rRNA level and from 0.5% to 8.0% at the 16S rRNA gene level throughout the incubation period. However, there was a clear time-dependent succession of different response groups underlying this steady increase, which was best evident at the 16S rRNA level (Fig. 4). Nine days after chitin amendment, 30 OTUs affiliated to unclassified bacteria, *Bacteroidetes*, *Spirochaetes*, *Proteobacteria*, *Fibrobacteres*, and *Chloroflexi* (decreasing number of OTUs) responded at the 16S rRNA level (Table S2). Two *Chitinivibrio* OTUs, OTU318 and OTU1405 (*Fibrobacteres*, 1.4% of all 16S rRNA copies), dominated this early response, followed by a pronounced decrease of their 16S rRNA relative abundance toward the end of the incubation time (Fig. 4). After 21 days of incubation, 68 additional OTUs responded at the 16S rRNA level. Besides the phyla mentioned above, these intermediate responders represented the *Acidobacteria*, *Ignavibacteriae*, *Parcubacteria*, *Firmicutes*, and *Cyanobacteria/Chloroplast* (decreasing number of OTUs). Transcriptionally most active intermediate responders were dominated by unclassified bacteria and members of the phyla *Bacteroidetes*, *Proteobacteria*, *Spirochaetes*, and *Chloroflexi* (in decreasing order), with none of the individual OTUs having distinctly more 16S rRNA copies than the others. The phyla that harbored responders after 43 days only were the *Verrucomicrobia*, *Planctomycetes*, and candidate phylum BRC1 (Table S2). However, in terms of relative 16S rRNA copy numbers, late responders were clearly dominated by the *Clostridium* cluster III OTU72 and OTU512 (*Firmicutes*), which together represented 4.7% of all bacterial 16S rRNAs. With the exception of members of the order *Myxococcales* (Deltaproteobacteria), which significantly responded at the 16S rRNA level only, the described succession at the 16S rRNA level was largely mirrored at the 16S rRNA gene level as well (Fig. 4).

### GlcNAc responders are clearly distinct from chitin responders.

**FIG 5:**
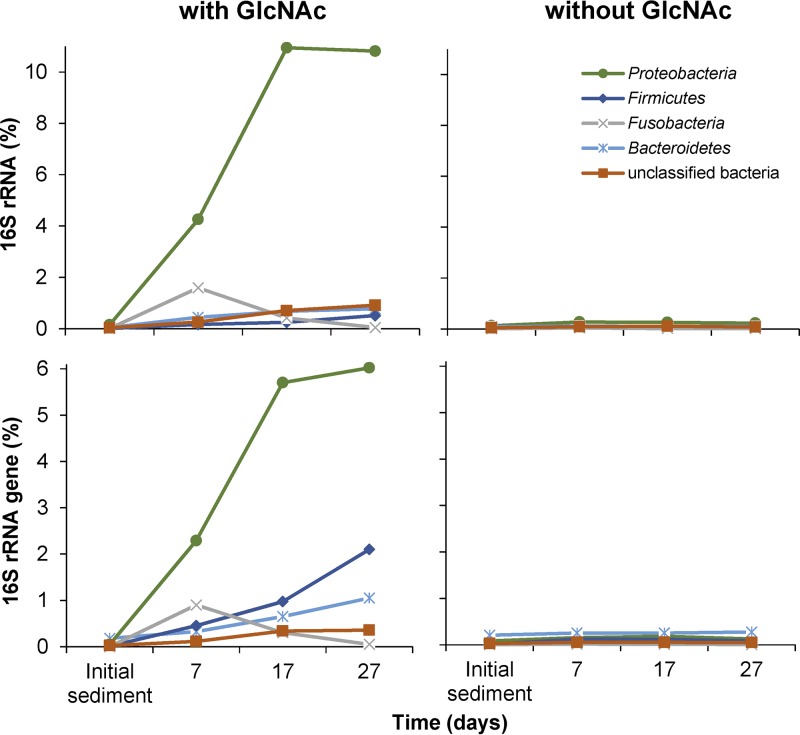
Temporal changes of OTUs (summarized by phylum) at the 16S rRNA level that significantly responded (FDR-corrected *P* < 0.05) to GlcNAc over time compared to the no-substrate control. Only phyla that corresponded in sum of their responding OTUs to ≥0.9% of bacterial 16S rRNA copies are shown for clarity. For details please refer to Table S3.

In the GlcNAc incubation line, 198 OTUs responded positively to regularly amended GlcNAc over time and were represented by 13 phyla and unclassified bacteria (see Table S3). During the total incubation period of 27 days, responding OTUs increased in sum from 0.3 to 13.8% at the 16S rRNA and from 0.5 to 10.3% at the 16S rRNA gene level (Table S3). Again, responses were strongest at the 16S rRNA level. However, in contrast to the chitin treatment, the overall response was driven by members of just one phylum, the *Proteobacteria* (Fig. 5). Here, 85 response OTUs affiliated to the *Proteobacteria* accounted for 4.3%, 10.9%, and 10.8% of all 16S rRNA copy numbers after 7, 17, and 27 days, respectively (Table S3). Among these OTUs, *Uliginosibacterium* OTU50 (*Betaproteobacteria*) clearly dominated the early response after 7 days (2.5% of total 16S rRNA copy numbers). After 17 days, *Azospirillum* OTU85 and unclassified OTU364 (both *Alphaproteobacteria*) as well as *Rhodocyclaceae* OTU66 (*Betaproteobacteria*) sharply increased in relative 16S rRNA copy numbers as well (1.1% to 2.1% per OTU) but were still less abundant than *Uliginosibacterium* OTU50 (2.9%). Only after 27 days, *Uliginosibacterium* OTU50 declined (1.4%) and was outnumbered in relative 16S rRNA copy numbers by *Azospirillum* OTU85 (3.3%) and *Rhodocyclaceae* OTU66 (2.3%).

Besides the *Proteobacteria*, 16 OTUs affiliated with *Bacteroidetes*, *Fusobacteria*, *Firmicutes, Spirochaetes*, unclassified bacteria, and *Cyanobacteria/Chloroplast* (decreasing number of OTUs) responded at the 16S rRNA level after 7 days of incubation as well (Table S3). Here, members of the *Fusobacteria* (OTU139, OTU344, and OTU9344) constituted the second most dominant response group after the *Proteobacteria* (1.6% of all 16S rRNA copies) but strongly declined thereafter again (Fig. 5). After 17 days of incubation, 35 additional OTUs responded at the 16S rRNA level and were affiliated with, besides the phyla mentioned above, the phyla *Verrucomicrobia*, *Acidobacteria*, *Chloroflexi*, and *Lentisphaerae* (decreasing OTU numbers). In terms of relative 16S rRNA copies, intermediate responders outside the *Proteobacteria* were dominated by unclassified bacteria and members of the phyla *Bacteroidetes*, *Fusobacteria*, and *Firmicutes* (in decreasing order), with none of the individual OTUs being distinctly more abundant than the others. Phyla that harbored responders after 27 days only were the phyla *Saccharibacteria* and *Parcubacteria*, and both were represented by just one OTU (Table S3). However, in terms of relative 16S rRNA copy numbers, late responders outside the *Proteobacteria* were dominated again by members of unclassified bacteria, *Bacteroidetes*, and *Firmicutes*, which showed an increasing response toward the end of the incubation time.

Among the positively responding OTUs in chitin-amended (203 OTUs) and GlcNAc-amended (198 OTUs) microcosms, only 23 OTUs responded under both treatments (7 *Bacteroidetes*, 2 *Chloroflexi*, 2 *Firmicutes*, 1 *Betaproteobacterium*, 1 *Epsilonproteobacterium*, 1 *Cyanobacterium*, 1 unclassified *Proteobacterium*, 1 unclassified *Parcubacteria*, and 7 unclassified bacteria) (see Table S4). None of these OTUs reached more than 0.27% (0.38%) of all 16S rRNA (gene) copies at any time point of the respective treatments (Tables S2 and S3). In addition, members of the phyla *Spirochaetes*, *Acidobacteria*, and *Planctomycetes* that responded to either chitin- or GlcNAc-amended treatments were represented by different OTUs within the respective phyla.

## DISCUSSION

Anaerobic chitin turnover in incubations of littoral lake sediment was readily observed without any delay (Fig. 2), which indicated the presence of indigenous microorganisms well adapted to this substrate in Lake Constance. The observed rates were well comparable to chitin turnover rates measured in aerobically incubated sediments and soils published previously (3). Depth-resolved chitinase activity, as measured by MUF-DC-hydrolyzing chitobiosidase, showed its highest activity in the upper oxic sediment layer and a decreasing hydrolysis rate in anoxic sediment with depth. This depth dependence was observed in other freshwater lakes as well (31) and might reflect, besides decreasing chitin availability, different polymer turnover strategies in oxic compared to that in anoxic environments. Aerobic bacteria typically secrete soluble exo- and endochitinases in concert with chitobiosidase to the environment that act synergistically on chitin hydrolysis to the monomer GlcNAc, which is then taken up by the cell (32, 33). In contrast, most anaerobic polymer-hydrolyzing bacteria take up oligomeric structures into the cell, where these are further depolymerized to mono- and disaccharides (12). Therefore, the employed enzymatic assay may have only captured extracellular chitobiosidase activity outside the cell, leading to an underestimation of the actual anaerobic chitin hydrolysis rate. This was reflected in the 4-fold higher chitin degradation rate in anoxic microcosms (4.4 nmol chitin h^−1^ g sediment [dry weight]^−1^), which was estimated by the release of total ammonium from the amino sugar, than the chitobiosidase activity in fresh anoxic sediment slurries.

Chitin was efficiently mineralized to CH_4_ (and CO_2_ [data not shown]), as was also evident from no transient accumulation of GlcNAc or possible fermentation intermediates with the exception of a small amount of acetate (Fig. 2). The electron balance indicated that approximately 60% of chitin degradation could be attributed to the methanogenic degradation network, which typically includes polymer hydrolysis, primary and secondary fermentations, and hydrogenotrophic and/or acetoclastic methanogenesis (34). Microorganisms involved in anaerobic respirations likely contributed to the remaining overall mineralization of chitin to CO_2_. In Lake Constance sediments, activities of nitrate- (35), Fe(III)- (36), humic acid- (37), and sulfate-reducing microorganisms (38–40) were previously observed.

Chitin degradation in anoxic sediment microcosms was linked to a continuous change of bacterial beta diversity over time (Fig. 3), which was attributed to a minor portion of the overall community (see PERMANOVA results). This succession of the transcriptionally active chitin-degrading bacterial community was also evident at the individual OTU level (Fig. 4). Three distinct phases were identified. The early phase (0 to 9 days) was clearly dominated by two *Chitinivibrio* OTUs, which represent members of the *Fibrobacteres*—a phylum that encompasses polymer hydrolysis as a phylogenetically widespread trait (23). The only cultured representative of the genus *Chitinivibrio* (Chitinivibrio alkaliphilus) is an obligately alkaliphilic bacterium isolated from an alkaline soda lake and strictly depends on the polymer chitin as growth substrate, which it ferments to H_2_, acetate, glycerol, ethanol, and formate (8). *C. alkaliphilus* grows by attaching to chitin, and cells lyse massively after chitin is degraded (8). This lifestyle conforms well to our observations with both responding *Chitinivibrio* OTUs, which responded strongly in the early phase followed by a complete breakdown of the population toward the end of the incubation. In addition, our results show that metabolically active representatives of the genus *Chitinivibrio* are not restricted to extreme environments such as alkaline soda lakes but also play an important role in chitin degradation in freshwater lakes.

The intermediate succession phase (9 to 21 days) of chitin degradation was characterized by a higher diversity of dominant responders, which represented not only the *Chitinivibrio* OTUs of the early phase but also unclassified bacteria and members of the phyla *Bacteroidetes*, *Proteobacteria*, *Spirochaetes*, and *Chloroflexi* (Fig. 4). The *Bacteroidetes* are known to harbor polymer-hydrolyzing members, which are active under aerobic and anaerobic conditions (41). However, since the classification of responding *Bacteroidetes* OTUs was restricted to the phylum level, little can be said regarding whether the responding OTUs were directly involved in chitin degradation or only indirectly by cross-feeding on the intermediate or metabolic end products of the primary hydrolyzing bacteria. *Proteobacteria*, especially members of the order *Myxococcales*, represented another dominant group of intermediate responders. Since members of the *Myxococcales* (suborder *Sorangiineae*) are known to degrade cellulose under oxic conditions (42), a primary involvement of the responding *Myxococcales* in chitin hydrolysis and degradation seems feasible. The responding members of the phylum *Spirochaetes* represent an interesting case. Although only one cultured member of the *Spirochaetes* has so far been observed to hydrolyze polymeric structures in pure culture (43), their association with hydrolyzing bacteria and the presence of chitinolytic enzymes in their genomes indicate that these microorganisms have an important impact during anaerobic polymer turnover (44, 45). Supporting this notion, a recent study showed compelling evidence that members of the spirochaetal genus *Treponema* are involved in hemicellulose degradation in the guts of wood-feeding termites (46). Last but not least, most of the intermediate responders that affiliated with the phylum *Chloroflexi* grouped within the family *Anaerolineaceae*, which has only four cultured representatives that can grow anaerobically on different monosaccharides as well as dimeric, oligomeric, or polymeric structures (47). Results of our study might also indicate an involvement in anaerobic chitin degradation.

The late succession phase (21 to 43 days) of chitin degradation was characterized by a complete breakdown of the responding *Fibrobacteres* and a strong increase of *Firmicutes* affiliated with the *Clostridium* cluster III. While the latter became the dominating late responders, members of the intermediate responders either increased further in their relative 16S rRNA (gene) copy numbers or stayed relatively stable (Fig. 4; see also Table S2 in the supplemental material). *Clostridium* cluster III was reclassified as the genus *Ruminiclostridium* (48, 49). *Ruminiclostridium* spp. contain many polymer-hydrolyzing members that can use cellulose, xylan, or chitin as carbon and energy sources (50–52). For example, Ruminiclostridium cellulolyticum, Ruminiclostridium cellobioparum, and Ruminiclostridium hungatei have all proven chitinolytic activity under anoxic conditions (52, 53). As such, the responding members of *Clostridium* cluster III (*Ruminiclostridium* spp.) were very likely directly involved in chitin hydrolysis and turnover in the late phase of chitin degradation in our incubations.

The specific microbial succession during anaerobic chitin degradation was corroborated by our control incubations using GlcNAc as the monomeric structure of chitin as the substrate. Here, OTUs that responded strongly under chitin amendment typically showed no significant response in terms of their 16S rRNA (gene) copy numbers. This indicated that these microorganisms clearly profit from chitin to a higher extent than from its monomer GlcNAc. This was especially true for OTUs of the genera *Chitinivibrio* and *Ruminiclostridium* (*Clostridium* cluster III), which are known to harbor chitinolytic species (8, 52) and were most responsive toward chitin but showed no response to the monomer GlcNAc. Vice versa, OTUs that strongly responded to the monomeric compound GlcNAc, mainly *Alphaproteobacteria* and *Betaproteobacteria*, showed no response toward chitin. This also indicated once more that, if anything, only a small amount of the monomeric compound GlcNAc is released into the environment during anaerobic chitin degradation. In addition, no other intermediate degradation products accumulated to a large extent during the incubation period. This reflects that the microorganisms involved in anaerobic chitin degradations act very efficiently together, resulting in a complete mineralization of this natural and important polymer. In summary, our results provide comprehensive insights into at which rate chitin is degraded under anoxic conditions and link this to a model of microbial succession associated with this activity.

## MATERIALS AND METHODS

### Sediment and lake water sampling.

Lake Constance is Central Europe’s third largest lake, situated in the prealpine region between Germany, Austria, and Switzerland. It has currently an oligotrophic status and a warm-monomictic mixing regime (54). Littoral sediment was sampled on 15 September 2015 for the setup of microcosm experiments and on 3 May 2017 for chitinase activity measurements. Sediment was taken from the littoral area of the Mainau Bay in Lake Constance (N47°42′7″, E9°11′43″), Germany. All sediment cores were sampled using push cores at a 2-m water depth with plastic tubes of 80-mm inner diameter. The sediment structure and layering were preserved in the cores, spanning a depth of 0 to 30 cm, and were covered with the overlaying lake water.

### Chitin hydrolysis rates in lake sediment.

Chitinase activity measurements were performed according to the procedure described by Köllner et al. (31). In brief, sediment samples were taken in triplicates from the surface as well as at 1, 2, 3, and 5 cm below surface. Samples (0.5 g [fresh weight]) of each layer were immediately transferred to 5-ml serum vials and flushed with N_2_ gas to provide anoxic conditions. Lake water overlaying the sampled sediment cores was filter sterilized with pore sizes of 5 μm and 0.1 μm (Merck Millipore Ltd., Tullagreen, Ireland) by a peristaltic pump (Ismatec Ecoline, Cole-Parmer, Wertheim, Germany), autoclaved, made anoxic, and treated with formalin (Merck, Schuchardt, Hohenbrunn, Germany) to a final concentration of 0.25%. Methyl-umbelliferyl-*N*,*N*′-diacetylchitobioside (MUF-DC; Sigma-Aldrich, St. Louis, MO) was dissolved in 100% dimethylformamide (DMF; Merck, Darmstadt, Germany). Sediment samples in 5-ml serum glass vials were amended with 1.5 ml of treated lake water and 25 μM MUF-DC (end concentration) under a constant flow of 100% N_2_. Preparations of the slurries with surface sediment were performed with the same procedure but under air. All slurries were incubated at 15°C for 23 h in the dark without agitation. After incubation, slurries were mixed by shaking and opened. To stop chitinase activity, 300 μl of the slurry was transferred to a 1.5-ml microcentrifuge tube and mixed with 30 μl ammonium-glycine buffer (0.05 M glycine, 0.2 M ammonium hydroxide, adjusted to a pH of 10.5 with NaOH). The mixture was incubated for 5 min at room temperature to allow for sedimentation of particles. The supernatant (200 μl) was then analyzed in a Varioskan Flash (Thermo Fisher Scientific, Waltham, MA) by applying an initial shaking step (600 rpm) for 5 s and thereafter a fluorescence measurement using an excitation at 360 nm and measuring the emitted fluorescence at 460 nm. To determine the dry weight content of the sediment, 1 g of fresh sediment was dried for 2 weeks at 60°C: 1 g (fresh weight) sediment corresponded to 0.62 g (dry weight) sediment.

Differences in observed chitinase activities were tested for significance (*P* < 0.05) using a one-factorial ANOVA and *post hoc* one-sided two-sample *t* tests in Microsoft Excel. Observed *P* values for the *t* tests were corrected for multiple testing using the Benjamini and Hochberg false-discovery rate method.

### Setup of microcosms.

Microcosms were set up on the same day as sediment sampling using the sediment layer 1 to 3 cm below the surface. For chitin and GlcNAc incubation lines, three sediment cores were sampled each, homogenized under a constant stream of 100% N_2_, and divided into 6 150-ml glass bottles by 50-g portions. Sediments were covered with 40 ml of anoxic filter-sterilized (0.2 μm) lake water that originated from the push core sampling, and the headspace was flushed with 100% N_2_ gas. Thereafter, microcosms were sampled for their initial sediment, sealed with butyl rubber stoppers, and preincubated for 7 days at 15°C to deplete internal substrates. After preincubation, each incubation line was split into two separate sets of triplicates. For the chitin incubation line, the first set of triplicates received 150 mg purified chitin once (setup “with chitin”). The second set of triplicates served as control without external substrates (setup “without chitin”). In the GlcNAc incubation line, the first set of triplicates received, every 3 to 4 days, a filter-sterilized (0.2 μm) GlcNAc solution (Fluka, Waltham, MA, USA) at a final concentration of ca. 400 μM (setup “with GlcNAc”). The second set of triplicates served again as control without external substrates (setup “without GlcNAc”) (see Fig. S1 in the supplemental material).

To ensure that chitin was the only external substrate supplied to the chitin incubation setup, commercially obtained chitin from crab shells (Sigma-Aldrich, Steinheim, Germany) was purified using the following procedure before application: 5 g of crab shell chitin was dissolved in 50 ml ice-cold HCl (25%) and stirred for 30 min. Thereafter, 250 ml of deionized autoclaved water was added, and the suspension was filtered through a 30- or 70-μm mesh (Franz Eckert GmbH, Waldkirch, Germany). The chitin remaining on the filter was transferred to a glass vial and washed once by the addition of 100 ml 50% ethanol (vol/vol). Thereafter, chitin was washed 4 times with 100 ml deionized autoclaved water and filtered through a paper coffee filter. Again, the chitin remaining on the coffee filter was transferred to a glass vial and washed again with deionized autoclaved water. Thereafter, the suspension was titrated toward neutral pH with 1 M NaOH. The suspension was filtered again through a paper coffee filter, and the chitin was removed from the filter and lyophilized.

GlcNAc concentrations and degradation products of chitin and GlcNAc were monitored for each microcosm every time the “with GlcNAc” setup replicates were supplemented with substrate. Gas samples from the headspace (200 μl) were monitored for accumulation of CH_4_ by gas chromatography (6000 Vega Series 2 GC; Carlo Erba, Italy), using a 45/60 Carboxen 1000 column (Supelco, Oberhaching, Germany) operated at 120°C and a thermal conductivity detector. One hundred percent nitrogen gas was used as carrier gas at a column pressure of 60 kPa. The injection port and detector were heated to 150°C and 180°C, respectively. Liquid samples (500 μl) were centrifuged (4°C, 14,000 × *g*, 5 min), and the supernatant was stored at −20°C until analysis. Before analysis, liquid samples were centrifuged again (4°C, 14,000 × *g*, 5 min) and the supernatant was used for measurements. Total ammonium (NH_3_ plus NH_4_^+^) concentrations were determined photometrically by a hypochlorite and phenol-based method (55). Formate, acetate, propionate, butyrate, lactate, and GlcNAc were monitored by high-performance liquid chromatography (HPLC; Shimadzu, Munich, Germany) with an Aminex HPX87H column (Bio-Rad, Munich, Germany) heated to 45°C and 10 mM H_3_PO_4_ as eluent at a flow rate of 1 ml min^−1^. Analytes were detected with a photodiode array detector at 200 nm (Shimadzu). The detection limit of all analyzed compounds was 10 μM.

Differences in endpoint product accumulation in substrate-amended treatments compared to that in their respective no-substrate controls were tested for significance using a two-sample *t* test assuming different variances in Microsoft Excel.

### Nucleic acid extraction and qPCR analysis.

Sediment in the microcosms was sampled at four different time points. First, samples were taken right at the onset of the experiment as described above (initial sediment). The other samples were collected after 9, 21, and 43 days of chitin amendment for chitin-amended and control microcosms in the chitin incubation line. For the GlcNAc incubation line, samples were taken after 7, 17, and 27 days after the first GlcNAc amendment, again both for substrate-amended and control microcosms. Sediment samples were frozen immediately in liquid N_2_ and stored at −60°C until further processing. RNA and DNA were extracted using the RNA PowerSoil Total RNA Isolation kit in combination with the RNA PowerSoil DNA elution Accessory kit (Mo Bio Laboratories Inc., Carlsbad, CA, USA). Remaining DNA in RNA extracts was removed with the TURBO DNA-free kit (Ambion, Thermo Fisher Scientific, Darmstadt, Germany). Reverse transcription of RNA into cDNA was performed using SuperScriptIII (Life Technologies, Darmstadt, Germany). Remaining RNA in DNA extracts was removed with RNase ONE (Promega, Mannheim, Germany). Thereafter, RNA and DNA were quantified using RiboGreen and PicoGreen (Life Technologies), respectively. Quantitative PCR (qPCR) of total bacterial and archaeal 16S rRNA genes was performed as described in reference 39.

### Amplicon sequencing and statistical analysis.

Bacterial 16S rRNA genes and cDNA (V3-V4 region) were amplified using the universal primer set 341F (CCT ACG GGN GGC WGC AG) and 802R (GAC TAC HVG GGT ATC TAA TCC) as detailed in reference 39. Sequence reads were quality controlled and subjected to *de novo* chimera filtering (UCHIME; Edgar et al. [56]) using Mothur v. 1.38.1 (57). This resulted in 19,421,489 high-quality reads, which clustered into 408,146 species-level OTUs (97% sequence identity). Each sample contained on average 16,581 ± 2,011 (mean ± standard deviation) OTUs. Taxonomic identity was assigned with the RDP Classifier (58) and the RDP 16S rRNA training set 16 using a confidence threshold of 0.80.

Alpha diversity was compared among samples by rarefying all replicates to an even sequencing depth in Mothur v. 1.38.1 (57). All further analyses were performed without rarefaction using R, version 3.3.2 (59). To filter against remaining sequencing artifacts and to enable meaningful statistical analysis, only OTUs detected by at least three amplicon reads in at least three different replicates were analyzed. Differences in beta diversity were analyzed using the weighted UniFrac distance (30) in combination with a principal coordinate analysis (PCoA) in the R phyloseq package 3.3.2 (60). In parallel, differences in beta diversity were tested for significance using a permutational analysis of variance (PERMANOVA) of the weighted UniFrac distances in the R vegan package 2.5.1 (61). The package edgeR 3.16.5 (62, 63) was used to test for significant (log fold change [logFC] ≥ 2, FDR-corrected *P* < 0.05) changes of relative OTU abundances according to both incubation time and incubation line.

### Data availability.

Amplicon data of 16S rRNA genes and 16S rRNA cDNA were deposited at the Sequence Read Archive at NCBI (64) under BioProject number PRJNA495895.

## Supplementary Material

Supplemental file 1

Supplemental file 2

Supplemental file 3

Supplemental file 4

Supplemental file 5
