# Supplementary material for: Microbial Succession of Anaerobic Chitin Degradation in Freshwater Sediments
Source: Appl Environ Microbiol. 2019 Aug 29;85(18):e00963-19. doi: 10.1128/AEM.00963-19 (PMC6715849; doi:10.1128/AEM.00963-19)
Supplement: Supplemental file 1 [file AEM.00963-19-s0001.pdf]

The microbial succession of anaerobic chitin degradation in freshwater sediments

Susanne Wörner[1, 2] and Michael Pester[1, 2, 3*]

1. Department of Biology, University of Konstanz, Universitätsstrasse 10, Konstanz, D-78457, Germany

2. Leibniz Institute DSMZ – German Collection of Microorganisms and Cell cultures, Inhoffenstr. 7B, D-38124 Braunschweig, Germany

3. Technical University of Braunschweig, Institute for Microbiology, Spielmannstrasse 7, 38106 Braunschweig, Germany

Supplemental Material

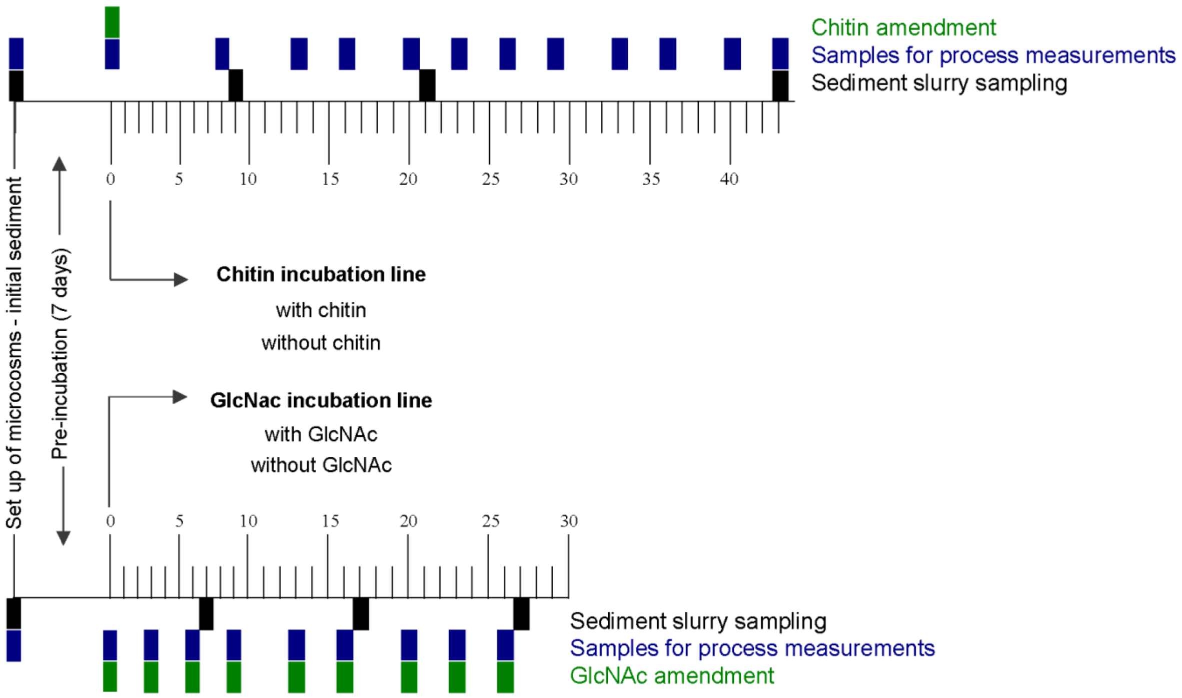

Figure S1. Overview of the experimental set up. Time points of substrate amendment are shown in green bars. Blue bars indicate sampling days for gas headspace (CH_4 and H_2) and liquid samples (GlcNAc, short-chained fatty acids, total ammonium). Black bars indicate sampling days for sediment slurries used for DNA and RNA extractions.

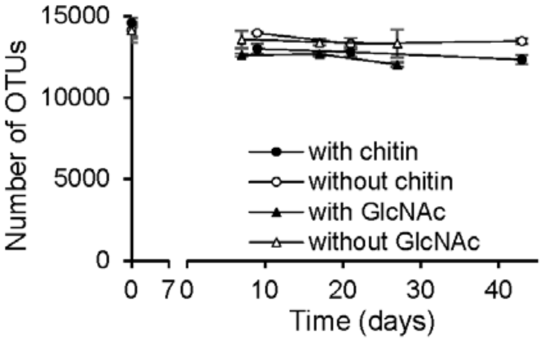

Figure S2. Time-resolved changes in the number of observed bacterial species-level OTUs in the individual treatments (16S rRNA gene analysis, 97% identity) when rarefied to an even sequencing depth of 68,826 reads per replicate (n = 3).

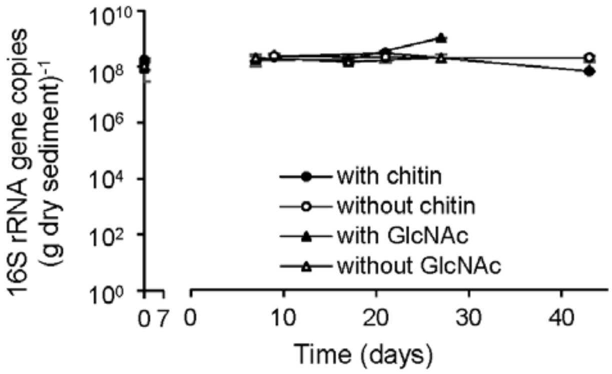

Figure S3. Abundance of total bacterial and archaeal 16S rRNA genes per gram sediment (dry weight) in the various microcosm setups as revealed by qPCR. The mean and one standard deviation are given, n = 3. Some error bars are smaller than the symbol size.

Table S1. (separate Excel file) Overview of the total bacterial community at the phylum level (for *Proteobacteria* class level) in the initial littoral sediment in the chitin (Table S1a) or GlcNAc (Table S1b) incubation line.

Table S2. (separate Excel file) Summary of all 16S rRNA (gene) OTUs at the approximate species-level (97% identity), which significantly responded (logFC > 2, FDR-corrected *p*-value < 0.05) to chitin amendment as compared to the initial sediment and the respective time point in the no-substrate incubation. For each OUT, the phylogenetic affiliation, the logFC, the average relative abundance (n = 3) in the individual treatments and time points at the 16S rRNA gene and 16S rRNA level as well as the representative sequence is given.

Table S3. (separate Excel file) Summary of all 16S rRNA (gene) OTUs at the approximate species-level (97% identity), which significantly responded (logFC > 2, FDR-corrected *p*-value < 0.05) to N-acetylglucosamine (GlcNAc) amendment as compared to the initial sediment and the respective time point in the no-substrate incubation. For each OUT, the phylogenetic affiliation, the logFC, the average relative abundance (n = 3) in the individual treatments and time points at the 16S rRNA gene and 16S rRNA level as well as the representative sequence is given.

Table S4. (separate Excel file) List of responding OTUs in chitin-amended microcosms that responded in GlcNAc-amended microcosms as well. Shown is the number of significantly responding OTUs at the respective phylum-level, for both 16S rRNA genes and 16S rRNA combined.
